# Understanding the experiences of lung cancer patients during the COVID-19 pandemic: a qualitative interview study

**DOI:** 10.1007/s11136-022-03283-z

**Published:** 2022-11-07

**Authors:** Sally Taylor, Melissa Stanworth, Charlotte Eastwood, Fabio Gomes, Binish Khatoon, Janelle Yorke

**Affiliations:** 1 Christie Patient Centred Research, The Christie NHS Foundation Trust, Wilmslow Road, Manchester, M20 4BX UK; 2 School of Nursing and Midwifery, The University of Manchester, Oxford Road, Manchester, M13 9PL UK; 3 Medical Oncology Department, The Christie NHS Foundation Trust, Wilmslow Road, Manchester, M20 4BX UK

**Keywords:** COVID-19, Lung cancer, Quality of life, Qualitative, Semi-structured interviews

## Abstract

**Purpose:**

The study explores experiences of lung cancer patients during COVID-19 and considers how changes to care delivery and personal lives affected patient needs.

**Methods:**

Semi-structured telephone interviews were conducted to explore experiences of lung cancer patients during COVID-19. Interviews were audio-recorded, transcribed verbatim and analysed thematically. Interview participants were purposively selected based on age, gender, treatment status, timing of diagnosis (pre/post first COVID-19 lockdown) from a sample of lung cancer patients (any histological subtype/any cancer stage/any point in treatment) who had completed a questionnaire exploring how participants’ lives were impacted by the pandemic and their thoughts on clinical care and remote communication.

**Results:**

Thirty lung cancer patients who participated in the questionnaire study were approached and participated in an interview. Three themes were identified: (1) Adapting to new modes of communication (focusing on experiences of remote communication); (2) Experience of care delivery during the pandemic (describing how all aspects of care delivery had been affected); (3) Impact of the COVID-19 pandemic on quality of life (QOL) (focus on the psychological impact and feeling of reduced support). Themes 1 and 2 are heavily interlinked and both had bearing on patients’ QOL experience.

**Conclusion:**

Lung cancer patients were impacted psychologically by changes to care delivery and changes in their personal life. The findings highlight some benefits to remote consultations but the stage of the treatment pathway and illness trajectory should be considered when determining if this is appropriate. Participants felt support from peers, family and friends was limited during the pandemic.

## Plain english summary

The COVID-19 pandemic had a huge impact on everyone’s lives. There are many factors which add to the vulnerability of lung cancer patients some of which may be specific to lung cancer patients (receiving active treatment) but some may be issues that would also affect the general population as well such as older age and the ability to engage with technology to communicate. The delivery of cancer services was affected during the pandemic which may have impacted lung cancer patients further. This work aims to understand how lung cancer patients have been affected during COVID-19 both in their personal lives and in relation to their cancer care. We interviewed 30 lung cancer patients to discuss their experiences. We will use patient experiences to address any problems with service delivery and improve patient experience. Patients discussed communication strengths and challenges, the impact on care delivery and the impact of COVID-19 on quality of life. Patient experiences highlight some benefits to telephone or video appointments, but this approach may not be suitable for everyone at every stage of their cancer journey. Patients highlighted the importance of support from family, friends and peers and how this was sometimes lacking during the pandemic. Better support options should be considered in the future.

## Introduction

The COVID-19 pandemic dramatically impacted peoples’ lives and healthcare services across the world. The National lockdown instigated by the United Kingdom (UK) government on 23 March 2020, imposed wide-ranging restrictions on freedom of movement and healthcare service delivery. Cancer services were particularly impacted [1, 2].

Around 48,000 people are diagnosed with lung cancer in the UK each year [3] and are usually treated with radiotherapy, systemic anti-cancer therapy (chemotherapy/immunotherapy) or a combination of both [4]. Lung cancer patients receiving any of these treatments are classed as ‘extremely vulnerable’ [5] according to a list created by chief medical officers in the UK and would be at an increased risk of contracting COVID-19. The list of extremely vulnerable people is not specific to lung cancer, it applies to many different diseases and conditions. There are other criteria within the list which are also likely to impact lung cancer patients such as age and the existence of comorbidities. 44% of all new lung cancer diagnoses are in people aged > 75 years [3] and many have common comorbidities such as Chronic Obstructive Pulmonary Disease adding to their vulnerability. The advice from Public Health England for ‘extremely vulnerable’ patients was to shield, meaning they should stay at home and minimise face-to-face contact [5]. Shielding is challenging and may prevent many patients from carrying out daily activities independently, limit their ability to exercise, and impact their mental health. Given that many lung cancer patients are older, they may be impacted by issues affecting the older population in general such as experiencing loneliness, or finding it difficult to communicate remotely. Research exploring the impact of COVID-19 on the cancer patient population have reported a decline in social, role and emotional functioning [6] and suggested that particularly during the first wave of the pandemic the emotional wellbeing of cancer patients was significantly affected [7]. A qualitative study exploring the impact of the pandemic on care delivery for surgical lung cancer patients also stressed the impact in terms of reduced social support and psychological distress [8]. Several studies have reported delays in cancer care as a result of COVID-19 [8, 9]. Any change to routine lung cancer management may cause further patient distress.

During the COVID-19 pandemic, lung cancer diagnosis and its subsequent management have been affected. These rapid changes reflected the shifting risk–benefit ratio for patients and diminished resources [10]. Reduction in outpatient visits and delays in treatment have been commonly reported [11]. Beyond modifications in treatment pathways, telephone consultations replaced many face-to-face appointments [12, 13]. The shift to models of virtual care delivery was the most common solution to care delivery issues during the pandemic [11], therefore the use of remote consultations has suddenly come to the forefront of clinical practice.

The study aims to explore the experiences of lung cancer patients during the COVID-19 pandemic and understand how changes to care delivery and their lives in general affected their physical, social and psychological needs.

## Methods

### Study design

This is a qualitative study using semi-structured interviews.

### Participant selection

This qualitative study was linked to a cross-sectional questionnaire study where participants who had completed the questionnaire were approached to take part in the interview. The aim of the cross-sectional questionnaire study was to understand how the COVID-19 pandemic impacted the experience of lung cancer patients in terms of care delivery in order to plan for post-pandemic care and to determine how these changes and the restrictions faced during the pandemic impacted the functional status (physical, social and psychological) of lung cancer patients. Patients of any lung cancer histological sub-type, any cancer stage and at any point in their cancer management pathway between diagnosis and discharge or end of life were eligible for this study. Paper questionnaires were sent to a random sample of non-small cell and small cell lung cancer patients receiving anti-cancer treatment or in active surveillance (follow-up) at a specialist cancer centre in the North West of England. Checks were performed before distributing questionnaires to ensure patients had not died and were not receiving end of life or best supportive care. Participants completed an informed consent form for the questionnaire study which included optional consent for an interview. The qualitative interview study was conducted in addition to the questionnaire study to provide context to questionnaire responses and to provide greater insight into participants experiences. Participants were purposively selected for interview based on key criteria: age (over/under 70); treatment status (active treatment/surveillance); time of diagnosis (pre COVID-19 (i.e. before 23rd March 2020)/post COVID-19); sex. We used the criteria for sampling as patient experience may differ according to the key characteristics therefore, we aimed to interview a representative sample across these groups. The consent form asked for permission to access their medical records which contained the required clinical and demographic data. We aimed to interview approximately 30–40 patients.

### Study procedure

The study was approved by London Surrey Borders Research Ethics Committee (20/LO/1014). After arranging a convenient time, interviews were conducted via telephone by a female researcher (BK (PhD), MS (BSc), CE (MSc)) with experience in conducting qualitative interviews with oncology patients. All researchers were trained on the study procedures before approaching participants. The interviewers had no prior contact with participants until the interviews were arranged. Participants were aware that interviewers worked within a research team at the cancer centre but had no other information about them. Interviewers were not responsible for any aspect of lung cancer care delivery. Interviews lasted 19 minutes on average. Data collection took place between 4th December 2020 and 25th March 2021. Interviews were audio-recorded and transcribed. Interviews were semi-structured using a pre-defined interview schedule which was developed by the research and clinical team (lung cancer consultants and lung cancer specialist nurses) and informed by the literature and Patient and Public Involvement and Engagement activities. The interview schedule explored: how participants had been affected by the pandemic; how clinical care has been affected; experience of new modes of communication; impact of changes on quality of care; any additional support needs. Questions such as what aspects of your life have been affected by the pandemic and how has your clinical care been affected were asked (Table 1).

**Table 1:** Interview schedule

Question	Prompts
Introduce self and explain the purpose of the interview, recording and consent process	
How do you feel you have been affected by the corona virus and the current situation?	What aspects of your life have been affected? How have aspects of your social life been affected? For example, hobby and leisure activities, relationships with family and friends How have these changes affected you emotionally or psychologically? Do you have any worries or concerns? e.g. financial concerns In relation to the corona virus or COVID-19, do you understand what the term shielding means? Do you understand the importance of shielding?
When you compare yourself with others from your age, would you say that health-wise you are doing as well, better or worse than them?	Do you consider yourself overall fit for your age? Do you think your daily life activities have been particularly more affected during this pandemic because you may be less fit and independent than perhaps others? What exactly do you think was particularly challenging for you to manage on your daily life?
How has your clinical care been affected?	What aspects of your clinical care have changed? Has your treatment been affected? Have you had fewer appointments? Have you been able to speak to the same health professionals you would do normally?
Do you think the level of communication with the hospital team (doctors and nurses) about your care has changed during this period?	Can you explain how the level of communication has changed? Did you find any new challenges or benefits with this communication?
Can you explain your experience of a telephone consultation?	Are there any positives or negatives of a telephone consultation compared to face to face? Have you experienced any problems with telephone consultations i.e. missing the call, communication or technology difficulties
How have the changes to clinical practice impacted on the quality of the care you have received?	Do you have any concerns about the care you have received? Can you describe these concerns? Do you feel you are getting the same level of care as you were before the COVID pandemic?
Thinking about your daily life, are there any areas where you feel you need additional support?	What kind of support would be helpful? Are you satisfied with the contact and support from the clinical team at The Christie? How could this support be improved?
End of interview Thank participant for their contribution	

### Data analysis

Data collection and analysis occurred concurrently. Data collection ceased when data saturation was reached (Saunders et al., 2018). Interview data were analysed using inductive thematic analysis [14] which involved: reading and familiarisation; generating initial codes, and using these to code each transcript; grouping codes into potential themes; reviewing and refining themes in relation both to individual transcripts and the whole transcript set; naming themes; and producing the report. The first three transcripts were coded independently by BK, CE and MS to create preliminary codes and developing themes. The codes and themes were continuously reviewed as the remaining transcripts were coded and themes iteratively revised to offer the most accurate and concise representation of the data. Initial codes were cross-referenced with theory and prior research. After creation of a comprehensive code list, the final phase involved defining and naming themes. At this stage, each researcher reviewed the codes individually in order to decide which codes qualitatively described similar issues and therefore could be grouped together as an overarching theme. Researchers created individual theme lists and these were then discussed amongst the group in order to reach a consensus. For example, the following codes: technology difficulties; experience with different modes of communication; advantages/disadvantages of telephone communication became theme 1. Throughout this process, codes were discussed and verified with the team to ensure credibility and trustworthiness.

## Results

**Fig. 1:**
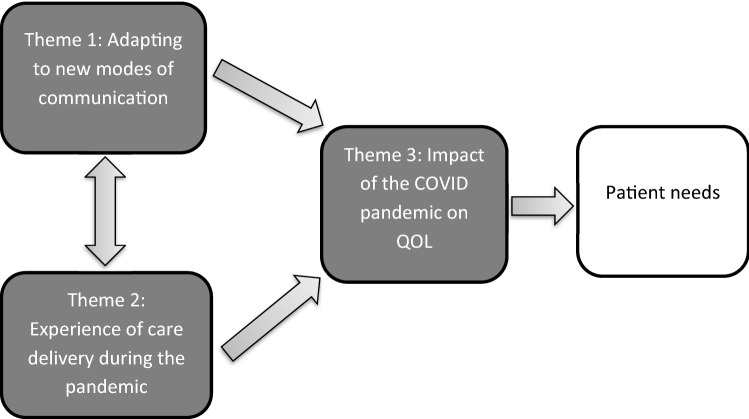
Themes identified from semi-structured interviews and how they interrelate with each other and feed into patient needs

Three hundred and five participants returned questionnaires, 184 agreed to be approached for interview and 30 were approached and interviewed. Key characteristics of interview participants are presented in Table 2. Three key themes were derived from the analysis: Adapting to new modes of communication; Experiences of care delivery during the pandemic; and Impact of the COVID-19 pandemic on quality of life (QOL). As presented in Fig. 1, Adapting to new modes of communication and Experiences of care delivery during the pandemic are heavily interlinked. The experiences of participants in terms of the new communication methods and changes to care delivery had a bearing on patients QOL experiences, for example, the changes in clinical practice were sometimes difficult for patients to deal with psychologically so this affected their QOL. In many cases, if a patients’ QOL was affected, then they often had needs that were not being met such as the need for improved communication or additional support. QOL is the key step between patient experiences and patient needs. Key differences between the responses of patients on active treatment and those in active surveillance or follow-up will be discussed throughout. Participant quotes are presented throughout with the following descriptors: LP01, Active/FU, PS0 (Lung patient 01, Active Treatment or Follow up, ECOG Performance Status 0–4). No differences were noted in terms of the other sampling criteria.

**Table 2:** Key characteristics of participants (*n* (%))

Sex	
Male	15(50)
Female	15(50)
Age	
Mean	70
Range	50–85
Treatment status	
Active treatment	17(57)
Active surveillance/follow up	13(43)
Cancer stage*	
1	3(10)
2	2(7)
3	11(37)
4	14(46)
ECOG Performance Status**	
0	10(33)
1	11(37)
2	7(23)
3	2(7)
Date of diagnosis	
Pre first COVID-19 lockdown	20(67)
Post first COVID-19 lockdown	10(33)

*Lung cancer stages define the size of a tumour and whether it has spread to other areas of the body. Stage 1 is the least severe and means the cancer is up to 4 cm. Stage 4 is the most severe and can mean the cancer is in both lungs, lymph nodes outside of the chest, in the covering of the lung or heart or the fluid around the lungs or has spread to other organs of the body

**ECOG Performance Status is a tool widely used to assess the functional status of a patient. Stage 0 is the least severe and means the patient is fully active and able to continue all pre-disease performance without restriction. Stage 4 is the most severe and means a person is completely disabled, is confined to a bed or chair and cannot carry out any self-care

### Adapting to new modes of communication

Patients reported positive and negative experiences of communication changes during the pandemic. Most patients on both active treatment and follow-up said they did not experience any technological difficulties associated with new communication methods. Problems reported were in the minority and were generally related to video calls rather than telephone calls.

‘No. We can’t do anything like that, we only can do things like that when my grandson’s here, you know? I don’t know technology like that.’ (LP6, FU, PS4)

Patient preferences for face-to-face or telephone consultations were mixed. There were no differences in preferences for mode of consultation delivery if patients were on active treatment or follow-up. Some patients preferred face-to-face consultations as they preferred to make eye contact. For some, their preference for face-to-face was linked to the information they were recieving in the consultation, patients felt it would be better to receive results, particularly if it was bad news, face-to-face. If a face-to-face consultation wasn’t an option, some patients would prefer video call to telephone as it would allow them to see facial expressions.

‘Maybe you should do a Zoom call, so you can at least see the consultant. Because facial expression passes out a lot of messages in my view, and that's missed on a telephone call.’ (LP22, Active, PS2)

Some patients preferred telephone consultations rather than face-to-face and a wide range of advantages and disadvantages were discussed by patients on active treatment and those on follow-up (Table 3). Some patients had no preference for either telephone or face-to-face consultations and were happy to be led by the clinical team as to the most appropriate option.

**Table 3:** Advantages and disadvantages of telephone consultations

Advantages of telephone consultations	
Improved communication	*‘I can’t see their facial reaction or anything, I’m more confident in saying, well, what does this mean and why can’t I do that, why can’t I take this vitamin.’ (LP17,FU, PS2)* *‘When the member of the team phones me, they’ve only got me to concentrate on.’ (LP1, Active, PS1)* *‘The level of communication has probably got better because we are only able to communicate *via* telephone at this moment in time.’ (LP5, Active, PS1)*
Psychological benefits	*‘I’m actually sat at home, I’m pottering around, I’m doing my own thing, so it distracts me a little bit and I’m not as anxious as I would have been sat in the waiting room in [the cancer centre]. So, from a mental health point of view, it’s better for me.’ (LP17, FU, PS2)* *‘I suppose the positive is not having to come into hospital. Part of me gets depressed when I come into the hospital because it’s a reminder’ (LP15, Active, PS1)* *‘When I’m on the phone I can manage to keep it together for the telephone conversation and then I’ve time to sit and think about what’s been said because I’m usually making notes while they are talking to me and it’s easier to digest then, for me.’ (LP17, FU, PS2)*
Practical benefits	*Well, obviously it minimises travel, it minimises travel to [the cancer centre]and all that entails.’ (LP18, FU, PS2)* *‘Well, they are usually within half an hour of the booked time. So it's not too bad.’ (LP20, FU, PS2)* *‘I’m sure they must feel the pressure down at [the cancer centre] when they look at the waiting room and they see how many people they’ve got to get through.’ (LP1, Active, PS1)*
COVID-19 implications	*‘You are not at risk in coming in contact with anybody who has got COVID.’ (LP25, FU, PS4)* *‘Why have a load of people going in and spreading this thing about. It's been bad enough, hasn't it.’ (LP29, FU, PS3)*
Disadvantages of telephone consultations	
Missing key elements you get from face to face	*‘No, the negative is that which I said, I prefer to look in the person’s eye, than not know who you’re talking to.…when you’re discussing something like stage three lung cancer and what the prognosis is.’ (LP30, FU, PS2)* *‘Face to face is okay, I don’t think we should lose that, but think it’s important for you to see your consultants and know who your team are, but positives, I would say, you can actually see your scan results there and then, you know, if you wanted to, you could be shown those.’(LP17, FU, PS2)* *‘They can…to me, actually hearing it face to face, I can take it in a bit more. I don’t know. I just feel better once I’ve seen the doctor.’ (LP23, Active, PS4)*
Informal and rushed	*‘Well like I say, they're varied. Some seem a little cold, as if oh I've got ten questions to ask you, I'll ask you them.’ (LP22, Active, PS2)* *‘Well, it’s just a telephone conversation, isn’t it? I mean, the info’s there, it’s just the personal touch isn’t there. Needs must in this time.’(LP4, FU, PS4)*
Situational issues related to taking the call at home	*‘There’s no-one there to support you. Yes, face to face, you’re there but you’re on the phone and you’re getting this information, there’s no-one there to help you.’ (LP19, FU, PS4)* *‘Well, I suppose human contact. I mean, in an ideal world, in the initial consultations my wife was with me, it helps to have someone else there to ask the questions that you forget to ask yourself.’(LP15, Active, PS1)* *‘It's very awkward because I live with my daughter who has got an autistic; I have got an autistic grandson, so he’s jumping about all the time, so it is very hard.’ (LP25, FU, PS4)*

At a time of limited face-to-face contact, communication with the clinical team and being kept informed was critical. Having regular contact with healthcare professionals was reassuring for patients and offered a sense of security. Many patients in both active treatment and follow-up talked positively about communication during the pandemic. Some felt communication had been more consistent and others felt more able to take the initiative to contact the clinical team rather than waiting for their appointment. Patients on active treatment really valued the support provided by the specialist nurses and felt they could contact them whenever they needed to. Patients on active treatment relied on this regular contact and found it difficult if information was lacking.

‘The specialist nurses are there if you need to speak to them but I can ring them up anyway and they will ring me back’(LP14, Active, PS3)

‘I don’t have any support from the clinical team… I just have that phone call once a month.’ (LP23, Active, PS4)

Patients on follow-up stressed the importance of being able to speak to the same health professional at every appointment. This continuity helped them to build a rapport and have the confidence to ask questions. Patients found communication difficult if they did not have continuity of care.

‘Seeing the same doctor each time, they get to know you, but that just doesn’t seem to happen’ (LP20, FU, PS0)

Patients on both active treatment and follow-up felt the appointment system could be improved as there were often problems with communication channels.

‘It just said telephone consultation Monday, and I didn't know whether it was am, pm, or anywhere near the sort of time’ (LP16, FU, PS4)

### Experiences of care delivery during the pandemic

Patients described their experience of care delivery during the pandemic. The discussions within this theme focused on patients’ experience of care delivery as a whole, not just at the cancer centre. Many patients did not feel the clinical care they had received at the cancer centre had been affected by the pandemic and spoke positively about their experiences. Some of the patients on follow-up felt the number of appointments they had received during the pandemic had reduced whereas those on active treatment did not feel the number of appointments had been affected. Some patients on active treatment however felt their diagnosis, treatment or scans were delayed due to COVID-19 and reported not having any face-to-face contact with the clinical team.

‘The fact that I haven’t seen a doctor whatsoever since I was diagnosed with terminal cancer.’ (LP23, Active, PS4)

A few patients discussed practical changes that had been put into place at the cancer centre due to COVID-19 such as reduced footfall, no visitors, changes to hospital entrances and screening for COVID-19. The impacts of these restrictions on patients were both positive and negative.

‘There’s not as many queues now because they’ve reduced footfall.’ (LP17, FU, PS2)

‘I suppose it’s impacted my wife a little bit, having to wait outside in the car park.’ (LP24, Active, PS3)

‘But the queues outside there were horrendous, and on the days when it was just lashing down with rain, that wasn't a very pleasant experience.’(LP16, FU, PS4)

Patients on active treatment talked about the impact COVID-19 had on additional support. Patients did not feel they had the same access to support as they would have pre-pandemic. Restrictions and changes to clinical care delivery made it more difficult to meet people in similar situations and share experiences.

‘If you’re sat in a clinic for gynaecological cancers then you’re going to meet other women that have got the same condition so you get talking to somebody so I guess that is the only difference.’ (LP5, Active, PS1)

‘I cannot access facilities, say go to the hospice for complementary therapies, I cannot access various things that I would be able to have done if COVID was not around.’ (LP5, Active, PS1)

Most comments about homecare were from patients on active treatment and their experiences were mixed. Some patients felt very satisfied with their care despite changes to the way the service was delivered.

‘I’ve had the home care team and they’re a hundred per cent can’t fault them.’ (LP1, Active, PS1)

‘They only say between 9:00 and 3:00 because they don’t know…someone might show COVID symptoms, so they won’t be seen that day, you know what I mean? Whereas prior to COVID…they give you maybe a bit of a narrower timeslot.’ (LP1, Active, PS1)

Patient experience was mixed in terms of the care they had received at other sites. Very few patients on either active treatment or follow-up spoke positively about their experience with primary care during the pandemic. Patients had problems contacting primary care providers and felt generally neglected and let down by the service. Patients had drastically changed their opinions of their General Practitioners (GPs) and felt they did not have the same relationships with their GPs anymore and could not rely on them to provide the same level of care they had received previously. Patients were also much less supportive of telephone consultations in the context of primary care.

‘You can’t see the doctor face to face. You go to the hospital, you’ll get seen face to face but you go to your GP, they don’t want to know. You get a phone call. It’s not the same.’ (LP19, FU, PS4)

The majority of patients said there were no changes to the level of communication and clinical care provided by secondary care facilities. This included positive experiences with the clinical team and support groups.

‘They’ve been amazing and so have…because I had the radiotherapy at [local hospital] and they were absolutely fantastic.’ (LP21, FU, PS3)

‘A, sort of, self-help group run by the one of the nurses there…And over this last…12 months, we've had two Office Team briefings, so we're still in contact with people, and we can see people.’ (LP22, Active, PS2).

### Impact of the COVID-19 pandemic on quality of life

Participants reported how COVID-19 had affected their QOL. Participants spoke about the emotional and psychological impact; financial concerns; social interactions, relationships, and lifestyle. Many patients had been affected emotionally and psychologically by COVID-19. Limitations on everyday lives and activities put strains on patients and their relationships and often left patients feeling frustrated, lonely and anxious.

‘I’m really frightened of catching anything.’ (LP2, Active, PS2)

‘I’d like for COVID not to exist then I could perhaps get out for lunch and things like that and sometimes I feel a bit angry about it…I’ve not got over long left and I would prefer if COVID wasn’t around I can come and go as I please while I feel fit.’ (LP14, Active, PS3).

Some patients were less affected than others and had tried to maintain a positive attitude throughout the pandemic. In some cases, patients felt there had been no impact on their social interactions and relationships either because their social contacts were limited anyway or because they had found other ways to interact and did not perceive the lack of physical contact to be a problem. Patients highlighted the importance of support from family and friends and the majority found the lack of social and physical contact very difficult. Follow-up patients seemed to focus more on the lack of contact with family whereas patients in active treatment described missing social interaction more broadly.

‘The worst one is we can’t really see our grandchild.’ (LP12, FU, PS3)

‘The fact we've got technology helps, but it…you do miss, you know, the cuddle, if you like, when you meet people.’ (LP22, Active, PS2)

Participants felt let down and angry by the behaviour of others who did not seem to follow government guidelines.

‘So angry at people. Because you do see them, and even if you go out with a mask on, or not a mask on, it depends, people still barge through you, so you can social distance, but then other people don't.’ (LP22, Active, PS2).

‘It’s other people that you see on TV and the ones out partying, you know. It’s quite upsetting.’ (LP3, FU, PS2)

In some instances, participants felt confused over shielding guidance as they received conflicting information from different sources.

‘It was confusing, yeah, because I was saying to my wife, well, they must think I can go out. And she said, don’t be silly, you’ve got lung cancer.’ (LP3, FU, PS0)

‘But there was no communication as far as I can see with the GP, so the GP hasn’t got me down as someone who’s been shielding.’ (LP15, Active, PS1)

It was not just the changes to the patients’ personal lives that caused social and psychological consequences. For many patients, the changes to the way their clinical care was delivered also affected them. Not being able to have a relative there for support during appointments or inpatient stays was difficult for many patients. The move away from face-to-face contact also caused a significant psychological impact for some patients and often left them feeling ‘sad’ or ‘neglected’.

‘That’s a big one, yes, having the support there. I think that’s a real one, not having your family and especially my wife.’ (LP19, FU, PS4)

‘I wasn’t able to have visitors in hospital because of COVID and that impacted on me greatly, psychologically.’ (LP5, Active, PS1)

‘I suppose the biggest thing is not being able to talk to your consultant face to face, I have telephone conversations….it is a bit frustrating.’ (LP22, Active, PS2)

Some patients commented on how well the changes had been dealt with and how every effort was made by staff to support patients. One patient felt having to go to appointments alone actually had a positive impact on her family.

‘When I started to go to [the cancer centre], it was great, I had my daughter with me… and then it stopped then when no one was allowed in, I was a bit worried, but it was marvellous after because they were so good to me, you know? So I’d no issues after that…the way everything was set up was marvellous.’ (LP11, FU, PS3).

‘So, I think, for me, it’s all to do with my mental wellbeing and my family. It’s less impact on them as well, you know, they can, sort of, carry on with their lives.’ (LP17, FU, PS2)

Several patients found COVID-19 impacted their lifestyle as they were unable to do normal activities. Patients were going out much less frequently than they used to and were relying on family and friends. The working lives, hobbies and activities of patients were impacted by COVID-19 with many saying holiday plans were affected. For some though, the changes brought about by COVID-19 meant they did not feel as though they were missing out due to their cancer.

‘I work in a supermarket, you know, on checkouts and self-scan. So, I’m rubbing shoulders with people, that’s why I can’t go back.’ (LP4, FU, PS4)

‘The thought of not going abroad for holidays was upsetting at first because that’s what we used to love doing but when everyone went into lockdown, everyone’s in the same boat as me.’(LP14, Active, PS3).

Generally, patients on active treatment and follow-up did not feel they were impacted financially during the pandemic, one patient commented that they felt they were probably ‘better off’. Some patients however did feel they had suffered a financial impact either directly or they had concerns about the financial impact on their family.

‘I did have because I wasn’t getting any money from anybody.’ (LP4, FU, PS4)

‘I’ll be leaving my wife behind, who is dependent on quite a bit of help.’ (LP9, Active, PS2)

‘I have worries about my children not being able to earn enough money. My son’s just been laid off.’ (LP3, FU, PS2)

## Discussion

Thirty patients participated in qualitative interviews to determine the impact of COVID-19 on clinical care and the wider impacts on lung cancer patients’ physical, social, and psychological wellbeing. Three key themes were evident from the data: Communication strengths and challenges; Experience of care delivery during the pandemic; Impact of the COVID-19 pandemic on QOL. Patients on active treatment and those on follow-up broadly discussed similar experiences although there were a few key differences. Communication strengths and challenges and experience of care delivery during the COVID-19 pandemic were heavily interlinked and the experiences of patients in both these areas had implications on their QOL.

One of the major changes during COVID-19 was communication pathways between patients and healthcare professionals. Telephone consultations became the main method of communication. Although telephone consultations and other remote monitoring have been used previously in clinical trials [15–17], they are rarely used in oncology clinical practice. Patient experience of remote follow-up was mixed and many advantages and disadvantages were discussed. Patients missed face-to-face contact but highlighted practical and psychological advantages such as reduced travel, protecting themselves from COVID-19 and avoiding hospital visit anxiety. A study of older cancer patients found that patients felt uneasy and hesitant about reduced face-to-face appointments [18]. Telephone consultations have been described in other research as a barrier to support and patients reported having to be more persistent to get the support they needed [19]. This finding was echoed in this study as patients felt information was often lacking. Some patients felt communication was better over the telephone as it seemed more focused whereas others felt it was informal and rushed. Other studies have reported that patients were generally satisfied with telehealth but also reported they would not want it to continue after the pandemic [20] suggesting that patients were supportive when there was no alternative but their opinions may change in the future.

Some patients on active treatment felt they needed more support from the clinical team. They also reported feeling as though they were missing out on peer support by not attending appointments in person. Lack of peer support was evident throughout the literature [19, 21]. Another major change for patients was being unable to take friends or family to appointments with them. As stated in the literature, this made patients feel as though support was lacking [22] and raised feelings of isolation, loneliness and distress [19].

Both follow-up and active patients noticed changes in clinical pathways. Follow-up patients felt they were having fewer appointments and some patients on active treatment felt there had been delays in their diagnosis, scans and treatment. The literature describes a number of cases where delays were experienced [23–26]. Delays or even concerns about potential delays have been reported as a significant cause of distress for cancer patients [22].

Follow-up patients emphasised the importance of continuity of care whereas this was not mentioned by patients on active treatment. Patients on active treatment may have had less opportunity to build a rapport with the clinical team. Other research has reported that patients starting the oncology journey felt there were challenges establishing care during the pandemic [21]. New patients in this study mentioned the disappointment of not seeing a clinician in person. The point a patient is at on the cancer disease and treatment trajectory is likely to significantly impact their thoughts on telephone consultations. As other research has highlighted, for telephone consultations, it is likely to be more difficult to establish a rapport during a new patient consultation or when breaking bad news [27].

The overarching theme running through the findings from this study is the support needs of participants and how these have been impacted during the pandemic. There are four components to social support: instrumental (practical tangible support with daily tasks or receipt of services); informational support (guidance and advice); emotional support (love, protection and care); and companionship support (a sense of social belonging). The findings from this study highlight how participants’ social support in each of the four components were compromised during the pandemic suggesting they may have unmet needs. A systematic review reported that higher levels of unmet needs cause a decrease in QOL [28] therefore it is important to address gaps in social support wherever possible.

Although rich information about individual participants experiences can be gained using qualitative methodology, it does have its’ limitations as data is only collected from a small number of participants. The findings of this study may be limited as participants were only recruited from one cancer centre, therefore findings may not be generalisable across the UK or internationally. Other cancer services may have adapted differently during COVID-19 and patients’ experiences may have differed.

The psychological impact caused by changes to care delivery and changes in a patients’ personal life are evident. The findings highlight key areas that could be considered for care delivery going forward. There are some benefits to a telehealth approach therefore some of these practices could be continued. It is important however to consider the stage the patient is at in the treatment and disease trajectory when considering if telehealth is appropriate. Ideally, patients should be given the opportunity to meet the clinical team in person initially and wherever possible, continuity of care should be maintained. The lack of peer support and support from friends and family at appointments was a big issue for patients therefore interventions should be considered to provide additional support to patients.

## References

[1] Richards M, Anderson M, Carter P, Ebert BL, Mossialos E (2020). (2020) The impact of the COVID-19 pandemic on cancer care. Nature Cancer.

[2] Lai AG, Pasea L, Banerjee A, Hall G, Denaxas S, Chang WH, Katsoulis M, Williams B, Pillay D, Noursadeghi M, Linch D, Hemingway H (2020). Estimated impact of the COVID-19 pandemic on cancer services and excess 1-year mortality in people with cancer and multimorbidity: near real-time data on cancer care, cancer deaths and a population-based cohort study. British Medical Journal Open.

[3] Lung cancer incidence statistics | Cancer Research UK. Retrieved November 4, 2022, from https://www.cancerresearchuk.org/health-professional/cancer-statistics/statistics-by-cancer-type/lung-cancer

[4] NICE (2019, March 28) Lung cancer: diagnosis and management. Retrieved November 4, 2022, from https://www.nice.org.uk/guidance/NG122

[5] Department of Health and Social Care (2020, November 4) Guidance on shielding and protecting people who are clinically extremely vulnerable from COVID-19 - GOV.UK. Retrieved November 4, 2022, from https://www.gov.uk/government/news/clinically-extremely-vulnerable-receive-updated-guidance-in-line-with-new-national-restrictions

[6] Alexander A, Fung S, Eichler M, Lehwald-Tywuschik N, Uthayakumar V, Safi SA, Vay C, Ashmawy H, Kalmuk S, Rehders A, Vaghiri S, Knoefel WT (2022). Quality of life in patients with pancreatic cancer before and during the COVID-19 pandemic. International journal of environmental research and public health.

[7] Ciążyńska M, Pabianek M, Szczepaniak K, Ułańska M, Skibińska M, Owczarek W, Narbutt J, Lesiak A (2020). Quality of life of cancer patients during coronavirus disease (COVID-19) pandemic. Psycho-Oncology.

[8] Teteh DK, Barajas J, Ferrell B, Zhou Z, Erhunmwunsee L, Raz DJ, Kim JY, Sun V (2022). The impact of the COVID-19 pandemic on care delivery and quality of life in lung cancer surgery. Journal of Surgical Oncology.

[9] Kirtane, K., Geiss, C., Arredondo, · Brandy, Aasha, ·, Hoogland, I., Chung, C. H., … Oswald, L. B. (2022). “I have cancer during COVID; that’s a special category”: a qualitative study of head and neck cancer patient and provider experiences during the COVID-19 pandemic. *Supportive Care in Cancer*. 10.1007/s00520-021-06773-x 10.1007/s00520-021-06773-x PMC8799415 35091844

[10] European Society for Medical Oncology. ESMO management and treatment recommendations in the COVID-19 era: Lung cancer. Retrieved 2022, November 4 from https://www.esmo.org/guidelines/cancer-patient-management-during-the-covid-19-pandemic/lung-cancer-in-the-covid-19-era

[11] Powis M, Milley-Daigle C, Hack S, Alibhai S, Singh S, Krzyzanowska MK (2021). Impact of the early phase of the COVID pandemic on cancer treatment delivery and the quality of cancer care: A scoping review and conceptual model. International journal for quality in health care.

[12] European Society for Medical Oncology. ESMO management and treatment recommendations in the COVID-19 era: Lung cancer. Retrieved 2022, November 4 from https://www.esmo.org/guidelines/cancer-patient-management-during-the-covid-19-pandemic/lung-cancer-in-the-covid-19-era

[13] The Royal College of Radiologists (2022, March 30). RCR advice on non-urgent and cancer imaging during the coronavirus pandemic. Retrieved 2022 November 4, from https://www.rcr.ac.uk/posts/rcr-advice-non-urgent-and-cancer-imaging-during-coronavirus-pandemic.

[14] Braun V, Clarke V (2006). Using thematic analysis in psychology. Qualitative Research in Psychology.

[15] Leeson SC (2017). Telephone follow up of gynaecological malignancies: time to rethink our postoperative care? BJOG. An International Journal of Obstetrics and Gynaecology.

[16] Hoek PD, Schers HJ, Bronkhorst EM, Vissers KCP, Hasselaar JGJ (2017). The effect of weekly specialist palliative care teleconsultations in patients with advanced cancer -a randomized clinical trial. BMC medicine.

[17] Beaver K, Williamson S, Sutton C, Hollingworth W, Gardner A, Allton B, Abdel-Aty M, Blackwood K, Burns S, Curwen D, Ghani R, Martin-Hirsch P (2017). Comparing hospital and telephone follow-up for patients treated for stage–I endometrial cancer (ENDCAT trial): a randomised, multicentre, non-inferiority trial. BJOG: An International Journal of Obstetrics & Gynaecology.

[18] Galica, J., Liu, Z., Kain, D., Merchant, S., Booth, C., Koven, R., … Haase, K. R. (2021). Coping during COVID-19: a mixed methods study of older cancer survivors. 10.1007/s00520-020-05929-5/Published 10.1007/s00520-020-05929-5 PMC7786158 33404813

[19] Drury A, Eicher M, Dowling M (2021). Experiences of cancer care during COVID-19: Phase 1 results of a longitudinal qualitative study. International Journal of Nursing Studies Advances.

[20] Palandri F, Bartoletti D, Giaquinta S, D'Ambrosio F, Auteri G, Sutto E, Catani L, Vianelli N, Cavo M (2021). Telemedicine in patients with haematological diseases during the coronavirus disease 2019 (COVID-19) pandemic: Selection criteria and patients’ satisfaction. British journal of haematology.

[21] Moran HK, Brooks JV, Spoozak L (2020). Undergoing active treatment for gynecologic cancer during COVID-19: A qualitative study of the impact on healthcare and social support. Gynecologic Oncology Reports.

[22] Cohen M, Yagil D, Aviv A, Soffer M, Bar-Sela G (2022). Cancer patients attending treatment during COVID-19: Intolerance of uncertainty and psychological distress. Journal of Cancer Survivorship.

[23] Edge R, Mazariego C, Li Z, Canfell K, Miller A, Koczwara B, Shaw J, Taylor N (2021). Psychosocial impact of COVID-19 on cancer patients, survivors, and carers in Australia: A real-time assessment of cancer support services. Supportive Care in Cancer.

[24] Moraliyage H, De Silva D, Ranasinghe W, Adikari A, Alahakoon D, Prasad R, Lawrentschuk N, Bolton D (2020). Cancer in lockdown: Impact of the COVID-19 pandemic on patients with cancer. The oncologist.

[25] Frey MK, Ellis AE, Zeligs K, Chapman-Davis E, Thomas C, Christos PJ, Kolev V, Prasad-Hayes M, Cohen S, Holcomb K, Blank SV (2020). Impact of the coronavirus disease 2019 pandemic on the quality of life for women with ovarian cancer. American Journal of Obstetrics & Gynecology.

[26] Swainston J, Chapman B, Grunfeld EA, Derakshan N (2020). COVID-19 Lockdown and its adverse impact on psychological health in breast cancer. Frontiers in Psychology.

[27] Burbury K, Wong Z-W, Yip D, Thomas H, Brooks P, Gilham L, Piper A, Solo C, Underhill C (2021). Australian capital territory, and 8 breast cancer trials, newcastle, and 12 border medical oncology and haematology. Albury, and Internal Medicine Journal.

[28] Cochrane A, Woods S, Dunne S, Gallagher P (2022). Unmet supportive care needs associated with quality of life for people with lung cancer: A systematic review of the evidence 2007–2020. European Journal of Cancer Care.

